# Incorporating Unstructured Patient Narratives and Health Insurance Claims Data in Pharmacovigilance: Natural Language Processing Analysis of Patient-Generated Texts About Systemic Lupus Erythematosus

**DOI:** 10.2196/29238

**Published:** 2021-06-29

**Authors:** Shinichi Matsuda, Takumi Ohtomo, Shiho Tomizawa, Yuki Miyano, Miwako Mogi, Hiroshi Kuriki, Terumi Nakayama, Shinichi Watanabe

**Affiliations:** 1 Real-World Data Science Department Chugai Pharmaceutical Co Ltd Tokyo Japan; 2 Risk Communication Department Chugai Pharmaceutical Co Ltd Tokyo Japan; 3 Foundation Medicine Business Department Chugai Pharmaceutical Co Ltd Tokyo Japan; 4 Biometrics Department Chugai Pharmaceutical Co Ltd Tokyo Japan

**Keywords:** social media, adverse drug reaction, pharmacovigilance, text mining, systemic lupus erythematosus, natural language processing, NLP, lupus, chronic disease, narrative, insurance, data, epidemiology, burden, Japan, patient-generated

## Abstract

**Background:**

Gaining insights that cannot be obtained from health care databases from patients has become an important topic in pharmacovigilance.

**Objective:**

Our objective was to demonstrate a use case, in which patient-generated data were incorporated in pharmacovigilance, to understand the epidemiology and burden of illness in Japanese patients with systemic lupus erythematosus.

**Methods:**

We used data on systemic lupus erythematosus, an autoimmune disease that substantially impairs quality of life, from 2 independent data sets. To understand the disease’s epidemiology, we analyzed a Japanese health insurance claims database. To understand the disease’s burden, we analyzed text data collected from Japanese disease blogs (tōbyōki) written by patients with systemic lupus erythematosus. Natural language processing was applied to these texts to identify frequent patient-level complaints, and term frequency–inverse document frequency was used to explore patient burden during treatment. We explored health-related quality of life based on patient descriptions.

**Results:**

We analyzed data from 4694 and 635 patients with systemic lupus erythematosus in the health insurance claims database and tōbyōki blogs, respectively. Based on health insurance claims data, the prevalence of systemic lupus erythematosus is 107.70 per 100,000 persons. Tōbyōki text data analysis showed that pain-related words (eg, pain, severe pain, arthralgia) became more important after starting treatment. We also found an increase in patients’ references to mobility and self-care over time, which indicated increased attention to physical disability due to disease progression.

**Conclusions:**

A classical medical database represents only a part of a patient's entire treatment experience, and analysis using solely such a database cannot represent patient-level symptoms or patient concerns about treatments. This study showed that analysis of tōbyōki blogs can provide added information on patient-level details, advancing patient-centric pharmacovigilance.

## Introduction

Pharmacovigilance, monitoring drugs during their product lifecycle to detect, assess, understand, and prevent adverse effects or other problems [1], is facing a challenge in refining its systems and regulations to accommodate increasing data volume and advancing data analysis techniques. A recent report suggests that it is necessary to broaden the scope of pharmacovigilance to enhance patient care and safety [2]. Since modern pharmacovigilance activities rely heavily on clinicians and upon the pharmaceutical industry, information on disease burden and psychology at the patient level is often difficult to capture from health care databases [3], though these patient data are essential for understanding disease.

To expand the scope of pharmacovigilance to patients’ viewpoints, it is necessary to include data sources that can be used to analyze patient situations. Several studies [4-6] have explored the use of web-based resources such as Twitter in pharmacovigilance to include patients’ viewpoints. Similarly, in Japan, we previously examined Japanese-language disease blogs (*tōbyōki*) as a resource for patient-generated data from the internet to augment pharmacovigilance [7]. In these blogs, we found that patients share information about adverse events, drugs, and distress due to adverse events. Such information can improve our understanding of disease epidemiology, treatment status, and burden by providing details that cannot be captured by existing health care data sources.

Although several studies [4,8] have reported the utility of patient-derived data from the web for pharmacovigilance, concern over the effect of irrelevant data (ie, noise) has led some researchers to recommend that these data alone should not be used to derive pharmacovigilance statistics [9]. Using data from additional sources is one way to minimize this effect. We believe that this methodology is important to achieve patient-centric pharmacovigilance, particularly in areas where disease management requires an understanding of both epidemiology and burden from patients’ perspectives.

Systemic lupus erythematosus is a complex, autoimmune disease; information from multiple sources should be considered in disease management. In Japan, limited epidemiological information on systemic lupus erythematosus is available [10]. Systemic lupus erythematosus has been reported to impair quality of life [11], and patients with systemic lupus erythematosus have suggested that the most important topics to consider in disease management are strengthening well-being and minimizing disease burden [12]. The objective of this study was to examine the value of the combined analysis of patient-derived text data and health care professional–derived data, in conjunction with natural language processing techniques, in implementing patient-centric pharmacovigilance for systemic lupus erythematosus.

## Methods

**Figure 1:**
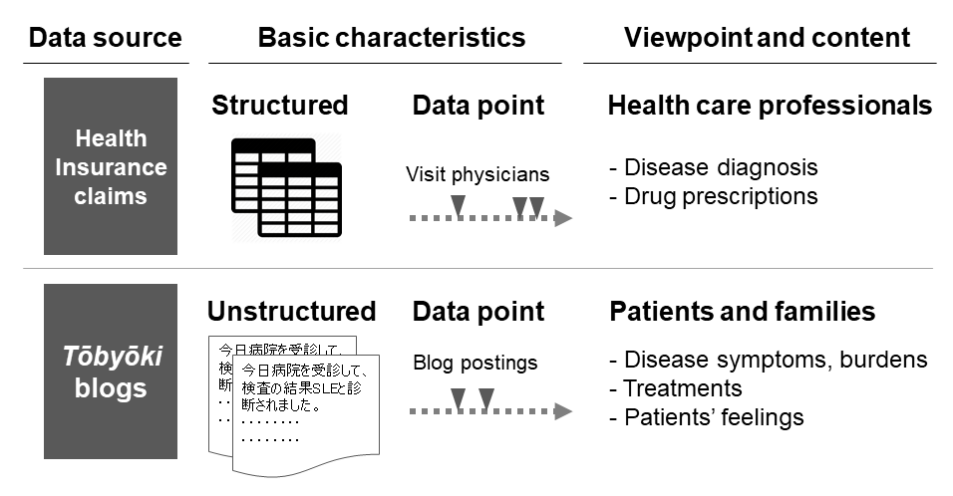
For each data source used in this study—health insurance claims data or tōbyōki blogs—basic characteristics such as data structures, data points, and contents are shown.

To understand epidemiology, treatments, and disease burden in patients with systemic lupus erythematosus, we analyzed 2 independent data sets: health insurance claims data and *tōbyōki* blog data. Each data set has its own advantages, therefore, using both provides greater insight on the epidemiology and burden of the disease (Figure 1). In this study, it was necessary to use each database according to its strengths; therefore, the analyses and results are presented separately for each point of focus.

### Ethics Statement

The study protocol was reviewed and approved by the Research Institute of Healthcare Data Science (RI2018008). The board waived informed consent because the data sources do not contain identifying information.

### Data Sources

#### Health Insurance Claims Data

We analyzed a Japanese health insurance claims database (JMDC Inc), which contains data from more than 3 million individuals enrolled in the database as of 2015. Patient data from January 1, 2015 to December 31, 2016 were extracted. International Classification of Diseases tenth revision [13] (ICD-10) codes were used to identify data from patients with systemic lupus erythematosus (ICD-10 code: M32); those with at least 2 claims with code M32, each in a different month, were extracted [10].

#### Tōbyōki Blog Data

As reported previously [7], we used a database of anonymous, publicly available *tōbyōki* blogs maintained by Initiative Inc. *Tōbyōki* is translated as *a diary-like account of a struggle with disease*. Each *tōbyōki* blog was manually checked to judge whether it was a *tōbyōki* blog or an irrelevant blog. *Tōbyōki* blogs were then manually tagged by disease (systemic lupus erythematosus or lupus nephritis) based on the blog’s title or introduction page. *Tōbyōki* blogs written in Japanese by patients with systemic lupus erythematosus or lupus nephritis between January 1, 2010 and February 7, 2018 were included in this study. To protect patient anonymity [14], all analysis results were output as summarized data and not individual-level data.

### Prevalence and Incidence of Systemic Lupus Erythematosus

#### Health Insurance Claims Data

Using the health insurance claims data, we identified patients with prevalent systemic lupus erythematosus, defined as systemic lupus erythematosus diagnosed between January 1, 2015 and December 31, 2016, and calculated the overall prevalence (with 95% CI), as well as by age and by gender. We also estimated the incidence (with 95% CI) by calculating the number of patients with incident systemic lupus erythematosus, defined as an initial diagnosis between January 1, 2015 and December 31, 2016 (no systemic lupus erythematosus diagnosis in the preceding 12 months) divided by the total population during both years.

### Systemic Lupus Erythematosus Treatments

#### Health Insurance Claims Data

Data from patients with systemic lupus erythematosus was used to identify medications during patients’ follow-up periods. Medications were coded according to the Anatomical Therapeutic Chemical classification system [15] or procedure codes, and data were summarized descriptively.

#### Tōbyōki Blog Data

Unstructured text written by patients was deconstructed into words using morphological analysis. Drug names mentioned in blogs were analyzed and summarized descriptively.

### Patient Complaints of Disease-Specific Symptoms

#### Health Insurance Claims Data

Symptom outcomes cannot be obtained from health insurance claims data.

#### Tōbyōki Blog Data

We explored patients’ skin abnormality and photosensitivity symptoms, which are characteristics of systemic lupus erythematosus [11]. Symptom terms were identified by corpus-based morphological analysis and summarized descriptively. We also performed word co-occurrence network analysis, as described previously [7], to map the occurrence of words in conjunction with specific known symptoms of systemic lupus erythematosus.

### Pain and Health-Related Quality of Life

#### Tōbyōki Blog Data

We assumed that *tōbyōki* blogs would contain descriptions of patients’ experiences and burdens during systemic lupus erythematosus treatment, which are difficult to assess using existing health care databases. To uncover such information from patients’ narratives, we applied natural language processing techniques. First, because we assumed that the first mention of a drug was the closest to the time the drug was prescribed, we identified those that contained mentions of typical drug therapies for systemic lupus erythematosus by searching the content of each blog. Then, we identified blogs that included any information from both before and after mentioning therapy for systemic lupus erythematosus by manually reviewing the blog contents to explore longitudinal changes and patient characteristics.

The number of pain-related words used in relation to systemic lupus erythematosus treatments was analyzed; term frequency–inverse document frequency (TF-IDF) analysis was conducted, which assigns a weight to each term based on the frequency of its occurrence in the document, to highlight the word characteristics for each text; a higher score may indicate that the term *x* is important for the document *y*.



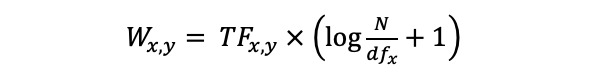



where *TF_x,y_* represents the frequency of term *x* in document *y*, *df_x_* represents the number of documents containing term *x*, and *N* represents the total number of documents.

We also sought to explore information on health-related quality of life from the unstructured patient narratives using the EQ-5D-5L questionnaire (EuroQol Group), which is a widely used validated instrument, consisting of 5 dimensions (mobility, self-care, usual activities, pain/discomfort, and anxiety/depression), for assessing health-related outcomes in both the general population and patients [16]. Based on the Japanese version of the EQ-5D-5L questionnaire [17], each dimension’s questionnaire items were manually reviewed to identify terms for that dimension. For instance, in the mobility dimension, for the statement “I have no problems in walking about,” we identified the terms “mobility” and “walking.” We (authors TO, ST, YM, MM, HK, and SW) independently identified related words, and discrepancies in the results were resolved by discussion.

### Data Analysis Tools

SAS software (version 9.4; SAS Institute) was used for data analysis. To process the unstructured text, we performed morphological analysis using MeCab [18], an open-source Japanese segmentation tool. Morphological analysis is commonly conducted to delimit words in text in which words are not separated by spaces, which is a characteristic of the Japanese language. R statistical software (version 3.6.2; The R Foundation) was used for text mining and data visualization.

## Results

### Study Population Characteristics

We analyzed health insurance claims data from 4694 patients with systemic lupus erythematosus and *tōbyōki* blog data from 671 patients with systemic lupus erythematosus. Health insurance claims data showed that systemic lupus erythematosus was more prevalent in females than in males (Table 1). More *tōbyōki* blog entries were written by females (634/671 patients, 94.5%) than by males (36/671 patients, 5.4%). The age distribution of patients represented in *tōbyōki* blogs was younger than that of patients represented by the health insurance claims data.

**Table 1:** Patient characteristics.

Age category		Health insurance claims data				*Tōbyōki* blog data			
		Total, n (%)	Male, n (%)	Female, n (%)	Total, n (%)		Male, n (%)	Female, n (%)	Unknown, n (%)
**All**		4694 (100)	994 (100)	3700 (100)	671 (100)		36 (100)	634 (100)	1 (100)
	≤19 years old	275 (5.9)	86 (8.7)	189 (5.1)	125 (18.6)		5 (13.9)	120 (18.9)	0 (0.0)
	20-34 years old	449 (9.6)	123 (12.4)	326 (8.8)	233 (34.7)		15 (41.7)	218 (34.4)	0 (0.0)
	35-49 years old	2175 (46.3)	337 (33.9)	1838 (49.7)	71 (10.6)		6 (16.7)	65 (10.3)	0 (0.0)
	50-64 years old	1557 (33.2)	379 (38.1)	1178 (31.8)	5 (0.7)		0 (0.0)	5 (0.8)	0 (0.0)
	≥65 years old	238 (5.1)	69 (6.9)	169 (4.6)	0 (0.0)		0 (0.0)	0 (0.0)	0 (0.0)
	Unknown	0 (0.0)	0 (0.0)	0 (0.0)	237 (35.3)		10 (27.8)	226 (35.6)	1 (100)

### Prevalence and Incidence of Systemic Lupus Erythematosus

**Figure 2:**
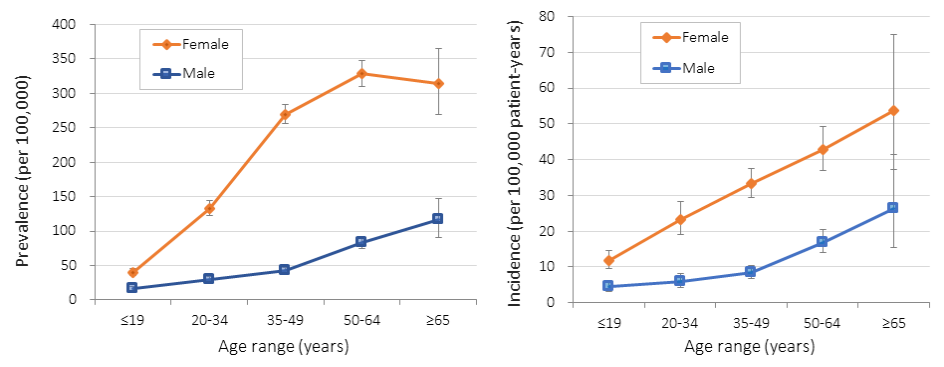
(A) Prevalence and (B) incidence of systemic lupus erythematosus for each age range, stratified by sex. Error bars represent 95% confidence intervals.

Using health insurance claims data, we found that the overall prevalence of systemic lupus erythematosus was 107.70 per 100,000 persons and was 4.4 times higher for females than that for males; females had a higher prevalence than males in all age groups (Figure 2). Similarly, the overall incidence of systemic lupus erythematosus was 16.86 per 100,000 person-years, and the incidences for all age ranges were higher for females than those for males.

### Systemic Lupus Erythematosus Treatments

Based on health insurance claims data, immunosuppressants, such as oral corticosteroids, and disease-modifying antirheumatic drugs were drugs frequently prescribed to patients with systemic lupus erythematosus (Table 2). Similarly, steroids, disease-modifying antirheumatic drugs, immunosuppressants, and therapeutic agents for osteoporosis were identified most frequently as drugs that patients mentioned (at least once) in *tōbyōki* blog data.

**Table 2:** Systemic lupus erythematosus drug treatments.

Drug treatments				Patients, n (%)
**Health insurance claims data (EphMRA ATC^a^ classification of drug [code])**				
	**All patients**			4694 (100)
		Oral corticosteroids, plain [H02A2]	2529 (53.9)	
		Proton pump inhibitors [A02B2]	1622 (34.6)	
		Antirheumatics, nonsteroidal plain [M01A1]	1432 (30.5)	
		All other antiulcerants [A02B9]	1333 (28.4)	
		Other immunosuppressants [L04X-]	1266 (27.0)	
		Bisphosphonates for osteoporosis and related disorders [M05B3]	1224 (26.1)	
		Vitamin D [A11C2]	1129 (24.1)	
		Nonnarcotics and antipyretics [N02B-]	1089 (23.2)	
		Topical antirheumatics and analgesics [M02A-]	1083 (23.1)	
		Systemic antihistamines [R06A-]	1023 (21.8)	
		Plain topical corticosteroids [D07A-]	891 (19.0)	
		Statins (HMG-CoA reductase inhibitors) [C10A1]	771 (16.4)	
		H2 antagonists [A02B1]	745 (15.9)	
		Expectorants [R05C-]	738 (15.7)	
		Angiotensin-II antagonists, plain [C09C-]	731 (15.6)	
***Tōbyōki* blog data (generic name of drug)**				
	**All patients**			671 (100)
		Steroid	499 (74.4)	
		Prednisolone	470 (70.0)	
		Loxoprofen sodium hydrate	220 (32.8)	
		Tacrolimus hydrate	190 (28.3)	
		Alendronate sodium hydrate	114 (17.0)	
		Aspirin	109 (16.2)	
		Acetaminophen	104 (15.5)	
		Lidocaine, Adrenaline bitartrate	101 (15.1)	
		Cyclophosphamide hydrate	99 (14.8)	
		Azathioprine	93 (13.9)	
		Alfacalcidol	89 (13.3)	
		Aztreonam	88 (13.1)	
		Calcium L-aspartate hydrate	83 (12.4)	
		Cyclophosphamide hydrate	82 (12.2)	
		Mycophenolate mofetil	80 (11.9)	

^a^Anatomical Therapeutic Chemical classification.

**Figure 3:**
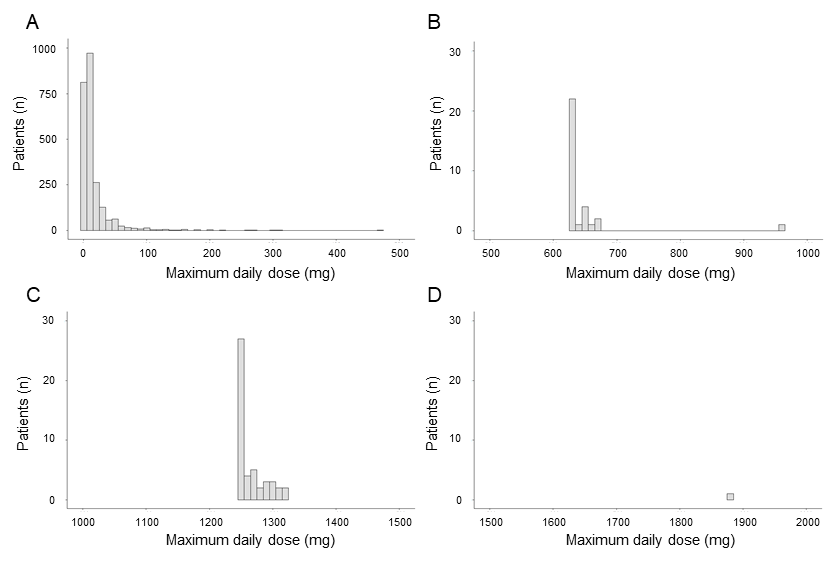
Distribution of the maximum daily dose of steroids: (A) 0-500 mg, (B) 500-1000 mg, (C) 1000-1500 mg, and (D) 1500-2000 mg.

For the steroids that, based on both data sets, were frequently used as treatments, we analyzed dose information using health insurance claims data (Figure 3). Among 2604 patients with systemic lupus erythematosus who had at least 1 prescription record for any steroid, 634 patients (24.3%) were prescribed 20 mg or more a day prednisone equivalent, 1844 patients (70.8%) were prescribed less than 20 mg, and 125 patients (4.8%) were prescribed an unidentified dose. The distribution of steroid dosages exhibited 3 peaks: the first, at 0.5-100 mg/day, seemed to represent maintenance therapy and the second and third peaks, at 625-675 mg/day and 1250-1325 mg/day, respectively, seemed to represent steroid pulse therapy.

### Patient Complaints of Disease-Specific Symptoms

Patient-level complaints that are not necessarily recognized as disease names cannot be derived from health insurance claims data. Symptoms that commonly present with systemic lupus erythematosus, such as “pain” and “feeling tired,” and some disease-specific symptoms, such as “moon face” and “arthralgia,” appeared frequently in blog text (Table 3). “Anxiety” and “stress,” which are neuropsychiatric symptoms associated with systemic lupus erythematosus, also frequently appeared. In addition, some terms representing conditions in patients’ lives, such as “pregnancy” (276/671, 47%) and “miscarriage” (69/671, 10%), appeared.

**Table 3:** Symptoms of systemic lupus erythematosus identified from tōbyōki blog data.

Symptoms mentioned in *Tōbyōki* blog data^a^			Patients, n (%)
**All patients**			671 (100)
	Pain	508 (75.7)	
	Symptom	504 (75.1)	
	Anxiety	498 (74.2)	
	Adverse drug reaction	495 (73.8)	
	Stress	467 (69.6)	
	Aggravation	430 (64.1)	
	Appetite	416 (62.0)	
	Headache	389 (58.0)	
	Shock symptom	386 (57.5)	
	Feeling tired	382 (56.9)	
	Recovery	354 (52.8)	
	Feeling itchy	326 (48.6)	
	Cough	322 (48.0)	
	Inflammation	297 (44.3)	
	Feeling abnormal	296 (44.1)	
	Swelling	296 (44.1)	
	Nausea	296 (44.1)	
	Moon face	295 (44.0)	
	Arthralgia	292 (43.5)	
	Slight fever	292 (43.5)	

^a^Number of patients who described each symptom at least once in their tōbyōki blog.

**Figure 4:**
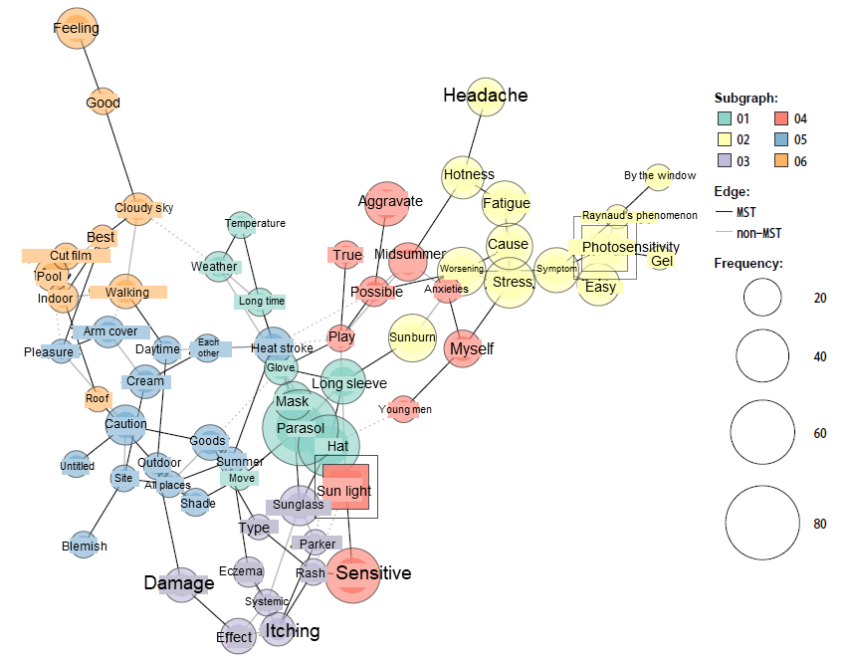
Network of words co-occurring with photosensitivity in tōbyōki blogs of patients with systemic lupus erythematosus. Because the original language of the blogs is Japanese, English translations are shown.

**Figure 5:**
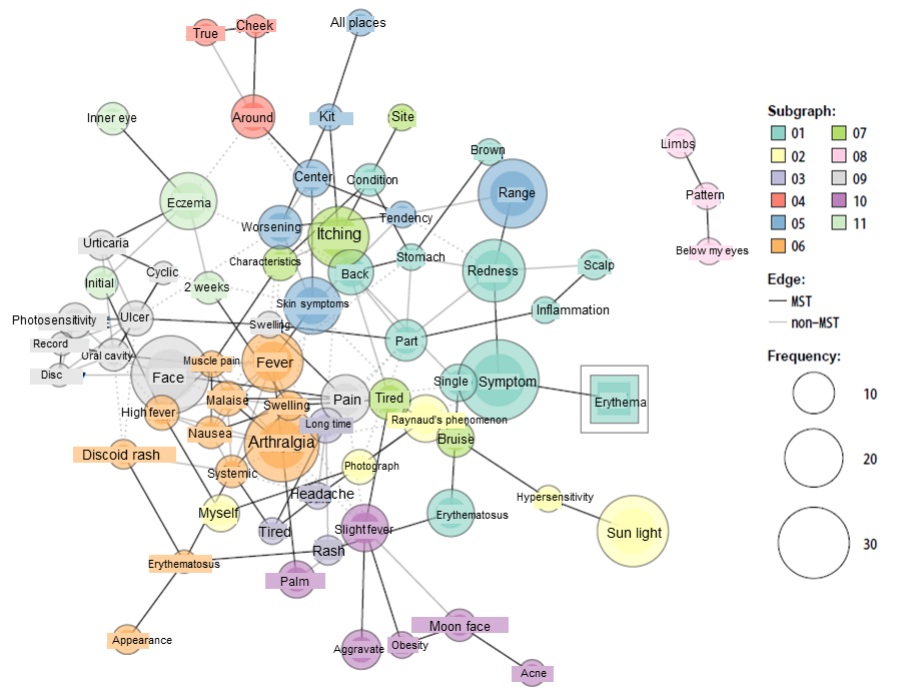
Network of words co-occurring with erythema in tōbyōki blogs of patients with systemic lupus erythematosus. Because the original language of the blogs is Japanese, English translations are shown.

We also conducted word co-occurrence network analysis to understand the characteristics of photosensitivity and erythema, which are 2 symptoms that are specific to systemic lupus erythematosus. In the word co-occurrence network analysis for photosensitivity (Figure 4), “photosensitivity” and “sunlight,” which were prespecified as central terms, were included in subgraph02 and subgraph04, respectively. Comorbid diseases and symptoms such as “Raynaud’s phenomenon,” “symptom,” “deterioration,” “headache” and “stressed” were observed. Other subgraphs did not connect directly; they included terms primarily related to “sunlight,” such as “sun protection goods” (subgraph01: “parasol,” “long sleeve,” “hat,” etc), “sun protection and symptoms” (subgraph03: “itching,” “rash,” “sunglasses,” “hoodie,” etc), “sun protection (location)” (subgraph05: “shadow,” “location,” etc), and “positive feeling” (subgraph06: “feeling,” “good,” “best,” “cloudy,” “indoor,” etc). The co-occurrence network of “erythema” (Figure 5) showed some symptoms as subgraphs. We classified subgraph themes as (1) skin and its color; (2) photosensitivity and its prevention; (3) symptoms of erythema; (4) cheeks; (5) degree and location of skin symptoms; (6) general symptoms of systemic lupus erythematosus; (7) itching; (8) appearance of hand, foot, and skin; (9) face and mouth symptoms; (10) other symptoms (moon face, fever, etc); and (11) timing of skin symptoms.

### Analysis of Pain and Elements in Health-Related Quality of Life

Among 671 *tōbyōki* blogs regarding systemic lupus erythematosus, 41 were identified as including information both before and after therapy was mentioned for systemic lupus erythematosus. Entries from the 41 blogs were divided into 3 periods: those from before mentioning therapy (number of pages identified: 10,064) those that were the first mention of therapy (number of pages identified: 515), and those from after mentioning therapy (number of pages identified: 109,977). TF-IDF analysis was conducted to examine changes in the number of pain-related words during the 3 periods.

**Figure 6:**
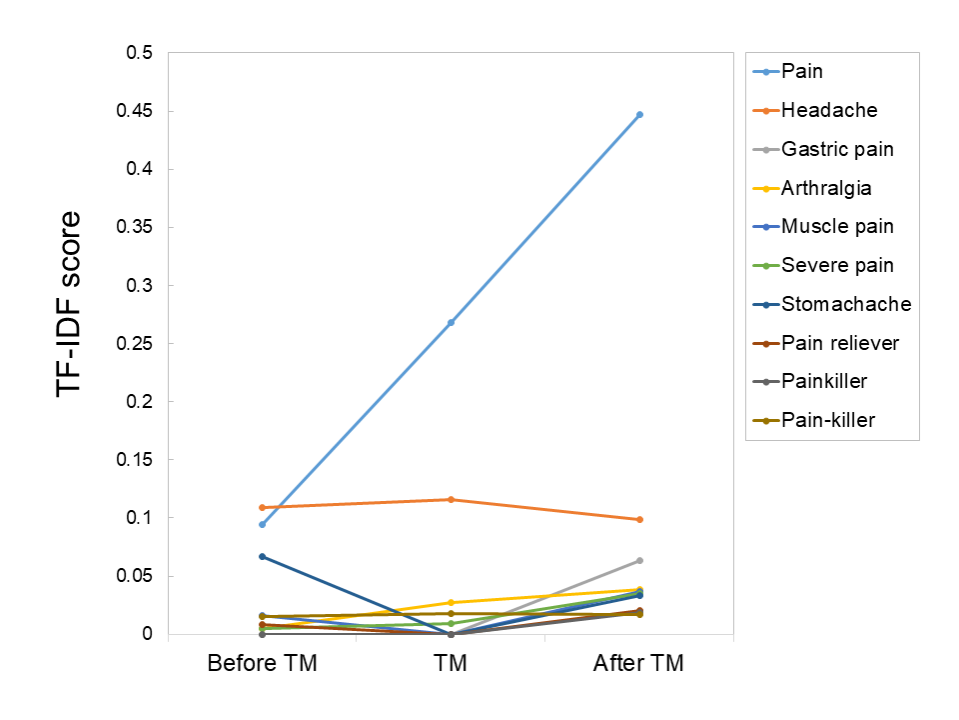
Changes in the importance of pain-related words before and after mentioning treatments. TF-IDF: term frequency–inverse document frequency; TM: therapy mentioned.

Pain-related words had higher TF-IDF values after therapy had been mentioned than those before therapy was mentioned (Figure 6). Symptoms with a large increase in TF-IDF (ratio >4) after therapy had been mentioned were pain, gastric pain, arthralgia, and severe pain; however, the TF-IDF for stomachache was less after therapy had been mentioned than that before (ratio ≤0.5).

**Figure 7:**
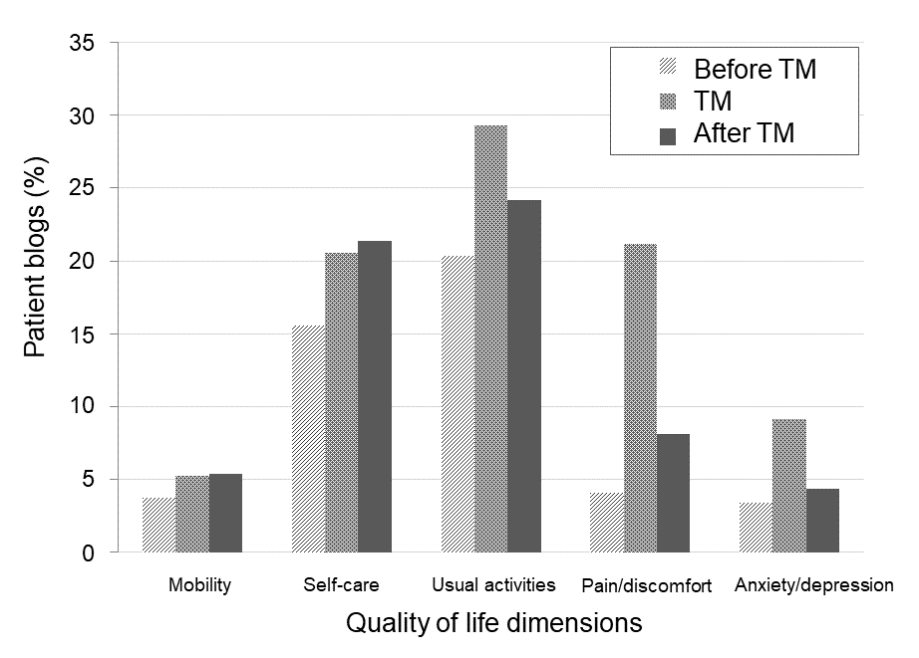
Health-related quality of life estimated from pre-specified keywords mentioned in tōbyōki blogs, corresponding to the 5 dimensions of the EuroQOL 5D-5L questionnaire. TM: therapy mentioned.

We examined the distribution of health-related quality of life words in the *tōbyōki* blogs (Figure 7). For all 5 dimensions, the frequency of words was higher when therapy was first mentioned or after therapy had been mentioned than that in the pretreatment period. For usual activities, pain/discomfort, and anxiety/depression words, the frequency of each term was highest when therapy was first mentioned, followed by the after therapy had been mentioned period and before therapy was mentioned. For mobility and self-care words, frequency increased from before therapy was mentioned period, when therapy was first mentioned, to after therapy was mentioned. Pain was most frequently mentioned when therapy was first mentioned (5.2 times more than before treatment was mentioned). Usual activities words were frequently mentioned in all 3 periods.

## Discussion

### Principal Results

In this study, analysis of *tōbyōki* blog and health insurance claims data facilitated comprehensive understanding of disease epidemiology, treatment, and patient burden. Although health care data sources such as health insurance claims data derived from health care professionals have played a central role in pharmacovigilance, these data do not include information about subjective symptoms or burden. Analyzing patient-written data sources using natural language processing techniques may be an efficient approach for gaining a more detailed understanding of patient burden for disease management.

The overall prevalence (Figure 2) of systemic lupus erythematosus was 107.70 per 100,000 persons and was 4.4 times higher among females, which are similar trends to those reported in the United States [19]. Systemic lupus erythematosus patients in Japan who have written *tōbyōki* blogs tended to be young. More patients represented by *tōbyōki* blogs were between 20 and 34 years old, whereas more patients represented by health insurance claims data were between 35 and 49 years old, suggesting that younger patients with systemic lupus erythematosus are more likely to write *tōbyōki* blogs than older patients. Given these differences in age distribution, *tōbyōki* blog data would be better suited for use in understanding disease burden in younger patients with systemic lupus erythematosus.

In both health insurance claims data and *tōbyōki* blog data, we found similar drug treatment trends (Table 2). Steroids appeared most frequently. Given that steroids are the standard treatment for systemic lupus erythematosus and that patients with systemic lupus erythematosus have a high burden of disease caused by adverse reactions to steroid treatment [20], the high frequency of steroid mentions in the blogs could reflect patients’ high attention to such treatment. A recent report suggests that immunosuppressive treatments for systemic lupus erythematosus remain poorly tolerated in some subsets of patients [21]; this high frequency of immunosuppressant mentions may also partly reflect patients’ worries about this type of treatment. Health insurance claims data cannot reflect the actual feelings of patients about their treatments. We revealed that the descriptions of a drug in *tōbyōki* blogs may be used to detect and evaluate burden, such as the patient’s attention and anxiety about the drug, which medical doctors and pharmaceutical companies do not always understand.

Although information on how the symptoms of the primary disease change (improve or deteriorate) with treatment and adverse events is vital in pharmacovigilance, it is impossible to obtain patient-level symptom information from health insurance claims data alone. In the clinical course of systemic lupus erythematosus, anorexia, general malaise, skin symptoms, and swelling of the face are known to occur [11]. Through the analysis of *tōbyōki* blogs, the sites where characteristic symptoms occurred could be estimated for example, “appetite,” “feeling tired,” “feeling itchy,” and “moon face” were identified, which were not obtained from health insurance claims data (Table 3). In addition, several terms related to physical appearance were also found, suggesting that patients may feel burdened by the negative effects of skin symptoms on their appearance (Figure 5). Thus, text data (patients’ blogs) enabled us to clarify patient-level symptoms and understand effects of treatments.

TF-IDF analysis showed that pain-related words became more important after the start of treatment than they were before the start of treatment (Figure 6). Headache and stomachache had lower scores after treatment than before treatment. This may suggest that some complaints voiced in daily life may be mentioned less frequently after treatment and may become less important than the primary disease. Since the TF-IDF score for gastric pain increased after treatment, it is likely that gastric pain may be an adverse event associated with treatment, since gastric pain would be mentioned similarly before treatment and during treatment if gastric pain was an effect of the primary disease.

In health-related quality of life data from *tōbyōki* blogs (Figure 7), since one of the main symptoms of systemic lupus erythematosus is pain, the frequency of pain-related expressions was high at the start of treatment. It is possible that pain was described as a motive for treatment, especially when first mentioning the treatment. The frequency of references to pain decreased after the start of treatment; it is possible that even as pain symptoms continued, patients gradually become accustomed to the pain, leading to a decrease in the frequency of blog mentions. Another possibility is that even if the pain continued, the descriptions used to convey the emotional feeling caused by the pain may change to another expression that was not captured in the analysis. The most frequently mentioned health-related quality of life dimension at any treatment point was usual activities, suggesting that patients are concerned about the influence of disease symptoms on these activities. The continuous increase in mobility and self-care descriptions may reflect increased attention to physical disability due to disease progression. Mentions of usual activities decreased after treatment compared to those during treatment, but the decrease (18% decrease) was less than that observed for pain (62% decrease) and anxiety/depression (53% decrease). This may also suggest that physical freedom decreases as the disease progresses. Health-related quality of life analysis would be difficult to perform using health care data alone. Our study indicated that health-related quality of life information could be inferred for the patient population based on the text information in *tōbyōki* blogs.

### Comparison With Prior Work

As a strength of this study, we applied several unique approaches to obtain effective insights from *tōbyōki* blogs. Although patient-level complaints can be expected in disease blogs, it is often difficult to quantitatively evaluate such information; therefore, qualitative evaluations such as word clouds and co-occurrence network diagrams are generally used. The unique approach taken in this study assumed that the first mention of a drug was the closest to the time the drug was prescribed. This assumption allowed comparisons between text data characteristics before and after a drug was mentioned. In pharmacovigilance, this approach could become an effective way to explore patient burden before and after treatment. Health-related quality of life is usually assessed through questionnaires administered when recruiting patients; however, this approach can be time-consuming and costly. In this study, we showed that *tōbyōki* blog data can contain health-related quality of life information and that it might be possible to identify elements related to health-related quality of life using text-mining approaches. Only a few studies have reported efforts to extract health-related quality of life information from unstructured patient-derived texts such as forums [22] or tweets [23]. A text-mining approach to patients’ unstructured data might also benefit the identification of psychological symptoms, which is difficult to measure using health care databases.

### Limitations

This study has several limitations. First, because *tōbyōki* blogs are written by only a segment of the patient population, generalization of the findings requires caution. For instance, older adults might be underrepresented in internet sources [24]. Second, as a patient’s condition becomes more severe, they may find it more difficult to continue writing their *tōbyōki* blog. This bias should be considered when interpreting the results. Third, text-mining analytics used in this study did not take into account dependency, syntax, and context in sentences. Thus, we did not distinguish between “pain has occurred” and “pain has disappeared,” and both would have been treated as a mention of pain. In future studies, analysis can be improved by using more advanced natural language processing techniques that can make this distinction. Last, we only used a single questionnaire (EQ-5D-5L) as an exploratory component of the analysis. Other disease-specific instruments for patients with systemic lupus erythematosus, such as PROMIS [25] or LupusQoL [26], should be investigated in future studies.

### Conclusions

A classical medical database represents only a part of a patient's entire treatment experience, and analysis using solely such a database cannot represent patient-level symptoms or patient concerns about treatments. This study showed that web-based text data from patients could add detailed patient-level information, which can be used to advance patient-centric pharmacovigilance.

## Abbreviations

TF-IDF: term frequency–inverse document frequency
